# Same-sex sexual behaviour among mammals is widely observed, yet seldomly reported: Evidence from an online expert survey

**DOI:** 10.1371/journal.pone.0304885

**Published:** 2024-06-20

**Authors:** Karyn A. Anderson, Julie A. Teichroeb, Malcolm S. Ramsay, Iulia Bădescu, Sergi López-Torres, James K. Gibb

**Affiliations:** 1 Department of Anthropology, University of Toronto, Toronto, Ontario, Canada; 2 Département D’anthropologie, Université de Montréal, Montréal, Québec, Canada; 3 University of Warsaw, Faculty of Biology, Biological and Chemical Research Centre, Institute of Evolutionary Biology, Warsaw, Poland; 4 Division of Paleontology, American Museum of Natural History, New York, NY, United States of America; 5 Department of Anthropology, Northwestern University, Evanston, Illinois, United States of America; University of Stirling, UNITED KINGDOM

## Abstract

Same-sex sexual behaviour (SSSB) occurs in most animal clades, but published reports are largely concentrated in a few taxa. Thus, there remains a paucity of published reports for most mammalian species. We conducted a cross-sectional expert survey to better understand the underlying reasons for the lack of publications on this topic. Most respondents researched Primates (83.6%, N = 61), while the rest studied Carnivora (6.9%, N = 5), Rodentia (4.1%, N = 3), Artiodactyla (2.7%, N = 2), and Proboscidea (2.7%, N = 2). Most respondents (76.7%, N = 56) had observed SSSB in their study species, but only 48.2% (N = 27) collected data on SSSB, and few (18.5%, N = 5) had published papers on SSSB. Of the unique species identified as engaging in SSSB in the survey, 38.6% (N = 17) have no existing reports of SSSB to the knowledge of the authors. In both the survey questions and freeform responses, most respondents indicated that their lack of data collection or publication on SSSB was because the behaviours were rare, or because it was not a research priority of their lab. No respondents reported discomfort or sociopolitical concerns at their university or field site as a reason for why they did not collect data or publish on SSSB. Multiple logistic regressions were performed to assess whether taxa studied, education level, or identification within the LGBTQ+ community predicted observing, collecting data on, or publishing on SSSB, but none of these variables were significant predictors. These results provide preliminary evidence that SSSB occurs more frequently than what is available in the published record and suggest that this may be due to a publishing bias against anecdotal evidence.

## Introduction

Same-sex sexual behaviour (SSSB) occurs in most animal clades and is defined as the act of engaging in sexual behaviours, such as mounting, intromission, and genital-oral or manual-genital contact with members of the same sex [1]. SSSB has been a focus of study in some primate and ungulate species (e.g., in deer (*Cervus spp*.) [2]; American bison (*Bison bison*) [3]; Japanese macaques (*Macaca fuscata*) [4, 5]) yet has remained a low research priority in the field of evolutionary biology.

This paucity of published reports may be explained by the perception that SSSB is a rare behaviour and is thus difficult to study systematically [6]. While systematic studies of animal behaviour did not begin until the 19th century, the notion that SSSB was rare, and therefore “unnatural” in the animal kingdom was used, and continues to be used, as evidence in debates of the ethics of human homosexuality [7, 8]. As attitudes and research on SSSB progressed throughout the 20^th^ and 21^st^ centuries, the notion of SSSB as a rare, “Darwinian Paradox” have persisted throughout the literature [6, 9–13], despite widespread reporting of SSSB across all major animal clades [1, 14–16].

Occasionally in animal behaviour research, behaviours which are perceived to be rare are actually found to be frequent when studied systematically [17]. There are several reasons why SSSB may be missed in the study of animal behaviour. Recent work on non-conceptive sexual behaviour in cetaceans found that the presence or absence of these behaviours was predicted by the number of articles published in each species, suggesting that non-conceptive sexual behaviours could be infrequently reported due to species research biases [18]. SSSB can occur in brief bouts that may overlap with other evolutionarily relevant sequences of behaviour, which poses a methodological issue when studying animal behaviour in the wild. Indeed, popular hypotheses for the adaptive value of SSSB involve its function in establishing and maintaining dominance hierarchies and social bonding [15, 19–21]. Thus, instances of SSSB may be grouped with other dominance or affiliative behaviours and are consequently not reported as their own distinct behaviours [22–26]. For this reason, many reports of SSSB could occur within research ancillary to SSSB, making these data difficult to access systematically through literature reviews [1]. Additionally, instances of SSSB may be misrepresented as opposite-sex sexual behaviour when visibility is poor, individuals cannot be identified, or the sex of the actors is assumed [1]. This may especially be the case when individuals are not identifiable, or for tree-dwelling species where visibility is limited. Finally, most of what is known about SSSB comes from opportunistic, anecdotal evidence, rather than systematic study [1, 15]. Anecdotes, which are narrative accounts of animal behaviour, have largely been replaced by quantitative and systematic data collection in animal behaviour research [with some exceptions, see 27, 28]. While calls for anecdotes have increased recently in some primatological journals, decades of bias against anecdotes could be influencing our current understandings of some behaviours that are perceived to be rare, such as SSSB [1, 28].

The aim of our study was to investigate observation and publishing trends on SSSB. We hypothesized that due to the methodological challenges mentioned above, as well as the perception that SSSB is rare, there may be a gap in the publication record on SSSB. We also sought to understand what factors may cause a publication gap if it exists. In addition to the methodological challenges of collecting data on SSSB, we hypothesized that reporting on SSSB may be biased by social factors that promote or inhibit a researcher’s interest and ability to publish on this topic. Researchers working in countries where homosexuality is criminalized may be less likely to, or unable to, publish papers on this topic if they wish to maintain good working relationships in that region. The political or social values of the institutions where researchers work may pose a barrier to their ability to publish on this topic. Conversely, researchers who identify as LGBTQ+ may be more likely to publish reports on SSSB, as the sexual, sex, and gender identities of researchers may impact the topics they study.

We conducted an expert survey among mammologists, wildlife biologists, and ecologists to assess whether researchers observe SSSB more often than they report in publications on their study species. Asking experts who work first-hand with their study species in the field is critically informative, given that the evidence base underlying much of what we know about the cross-species distribution of SSSB in mammals may be the product of research ancillary to SSSB, such as studies on dominance or affiliative interactions. Also, whether the lack of reported evidence of SSSB for particular species is actually evidence for its absence is a question that has remained largely unexplored.

Expert surveys may be a useful tool for wildlife biologists in the study of behaviours that are not frequently reported or perceived to be rare. Expert surveys have been utilized frequently in the social sciences to access the specialized knowledge of researchers [29] but have also been used within ecology and conservation biology [30, 31] and in studies of animal behaviour [32]. An expert survey was the only method that allowed us to access data across many species outside of the published record.

## Material and methods

We conducted an online expert survey using Google Forms [for more information on expert surveys, see 29]. Survey questions were created and reviewed by all co-authors for the purpose of this survey. A soft launch of the survey was sent out to three mammologists, prior to the initial recruitment for the survey, for testing and feedback. The survey was modified on the second day that it was live to provide clarity on one question (in Question 4, in a definition of same-sex sexual behaviors, “mounting” was changed to “sexual mounting”). The definition of SSSB is constantly evolving, though the typical definition is any genital contact between members of the same sex [14, 15]. This could include same-sex mounting, genital touching, oral-genital contact, and genital-genital contact. We acknowledge that, particularly in the case of mounting, some respondents may feel that not all mounting is sexual. We changed our wording to specify that only mounting which the respondents perceived to be sexual should be included. While the definition used in our survey is common within SSSB research, we recognize that there are issues with this definition. For example, not all genital touching may be sexual, as genital touching could be grooming, or otherwise non-sexual in nature. Alternatively, our definition may be too limiting. Recent work on SSSB has expanded the definition to include other social behaviours, such as courtship, pair-bonding, and alloparenting [16, 33].

The target population of our survey were individuals who identify as experts in the fields of wildlife biology, primatology, zoology, mammalogy, and/or associated fields. Participants were recruited via email invitations sent on university department listservs, organizational listservs, and sent to specific individuals. Links to participate in the survey were also distributed on Twitter to reach a larger audience. We asked participants for information on their study species, study sites, the sexual behaviour of their study species, and information on their data collection and reporting of same-sex sexual behaviour in their study species (see S1 Appendix for Survey Questionnaire). The survey did not ask for respondents to report on the frequency of the behaviours observed, but only whether they had been observed. Data were collected between June 1—August 31, 2021. Participants had the option to include their names and email addresses to be contacted for future phases of the study. Observations of both wild and captive animals were recorded, but we did not distinguish between these conditions in the survey questions. Our target sample size for the survey was 50–200 answers, which we based on prior expert surveys [29, 31, 34–36]. A sample size closer to 50 responses can tell us some preliminary information about data collection and publication trends, while a sample size closer to 200 would provide us with a more comprehensive understanding of data collection and publication on SSSB.

As a part of this research, we were interested in whether researchers who identify as broadly LGBTQ+ (Lesbian, Gay, Bisexual, Transgender, Queer, and other identities of sex, gender, and sexuality) are more or less likely to report on same-sex sexual behaviour in their study species. We included an optional question asking participants whether they identify broadly as LGBTQ+. Multiple logistic regressions were used to determine whether identification as LGBTQ+, as well as taxon studied and terminal degree obtained predicted observing, collecting data on, or publishing on SSSB. This research was approved by the University of Toronto ORE: RIS Protocol Number 00040675.

## Results

We received 65 total respondents to the survey, with some respondents reporting on multiple species. This resulted in a total of 73 responses across 52 different species (Table 1). Most respondents had obtained or were working towards a PhD (N = 44, 67.7%) in the fields of Anthropology, Biology, Psychology, Primate Behaviour or Conservation, or other related fields. 18.5% (N = 12) of our respondents indicated a master’s degree as their terminal degree, 9.2% (N = 6) indicated a bachelor’s degree as their terminal degree, and 4.6% (N = 3) did not specify. Most respondents (N = 49, 75.3%) were associated with a university as a professor, lecturer, post-doctoral researcher, graduate student, or research assistant. 18.5% (N = 12) of respondents worked in government, as zoo employees, or as project directors for non-governmental organizations, and 6.2% (N = 4) of respondents were unemployed or retired.

**Table 1:** Species reported to engage in SSSB in our expert online survey.

Order	Scientific Name	Number of accounts in survey	Existing literature
Artiodactyla	*Ornicus orca*	1	[37]
Artiodactyla	*Pecari tajacu*	1	[15, 38]
Carnivora	*Canis familiaris* (or, *Canis lupus familiaris*)	2	[15, 39–42]
Carnivora	*Mungos mungo*	1	
Carnivora	*Nasua nasua*	1	
Carnivora	*Panthera leo*	1	[15, 43–46]
Primates	*Alouatta guariba*	1	
Primates	*Ateles geoffroyi*	3	[47]
Primates	*Ateles sp*.	1	[47]
Primates	*Brachyteles hypoxanthus*	1	[48, 49]
Primates	*Cebus capuchinus*	1	[14, 48, 49]
Primates	*Cebus imitator*	1	
Primates	*Cercocebus sanjei*	1	
Primates	*Cercopithecus mitis stuhlmanni*	1	
Primates	*Chlorocebus sabaeus*	1	
Primates	*Colobus angolensis ruwenzorii*	1	
Primates	*Colobus vellerosus*	2	[50]
Primates	*Lagothrix flavicauda*	1	
Primates	*Lagothrix lagotricha poeppigii*	1	
Primates	*Macaca arctoides*	1	[14, 15, 51–58]
Primates	*Macaca assamensis*	1	
Primates	*Macaca fascicularis*	1	[14, 15, 59–61]
Primates	*Macaca mulatta*	8	[14, 15, 62–71]
Primates	*Macaca nemestrina*	1	[72–77]
Primates	*Microcebus murinus*	1	
Primates	*Pan paniscus*	1	[14, 15, 78–81]
Primates	*Pan troglodytes* (including one count in *Pan troglodytes verus*)	4	[14, 15, 82, 83]
Primates	*Papio ursinus*	1	[14, 15, 84–86]
Primates	*Pongo abelii*	1	[15, 87]
Primates	*Pongo sp*.	1	[14, 15, 87–89]
Primates	*Saimiri sciureus*	2	[90–95]
Primates	*Sapajus libidinosus*	1	[23, 96]
Primates	*Sapajus nigritus*	1	
Primates	*Sapajus sp*.	1	[23, 33, 96]
Primates	*Semnopithecus schistaceus*	1	
Primates	*Symphalangus syndactylus*	2	[14, 15]
Primates	*Varecia variegata*	1	[14]
Proboscidea	*Elephas maximus*	1	[97–100]
Proboscidea	*Loxodonta africana*	1	[15, 101–104]
Rodentia	*Fukomys darlingi*	1	
Rodentia	*Fukomys mechowii*	1	
Rodentia	*Xerus inauris*	1	

**Fig 1:**
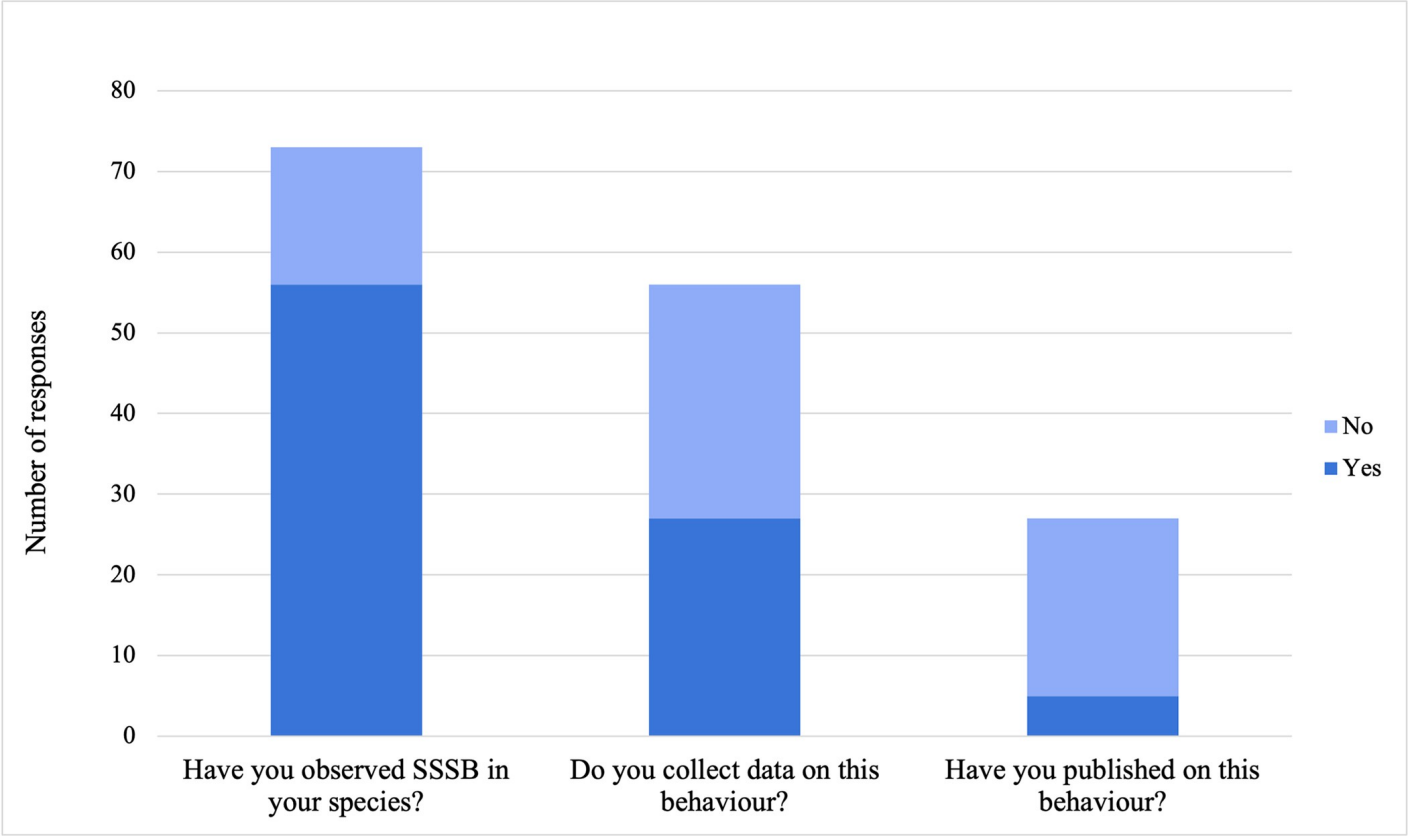
Responses of observations, data collection, and publishing on same-sex sexual behaviour in our expert online survey.

Most respondents studied Primates (N = 61, 83.6%), while the rest studied species within Carnivora (N = 5, 6.9%), Rodentia (N = 3, 4.1%), Artiodactyla (N = 2, 2.7%), and Proboscidea (N = 2, 2.7%). 76.7% (N = 56) of respondents indicated that they had observed SSSB in their study species, but only 48.2% (N = 27) indicated that they collected data on these behaviours (Fig 1).

Overall, we received 58 reports of SSSB across 42 of the 54 individual species identified. Thus, SSSB was reported in 77.8% of species studied by our respondents. Species identified as engaging in SSSB by survey respondents were compared with existing literature on SSSB in mammals (Table 1). Of the 44 unique species identified as engaging in SSSB in the survey, 17 (38.6%) have no existing published reports of SSSB to the knowledge of the authors.

**Fig 2:**
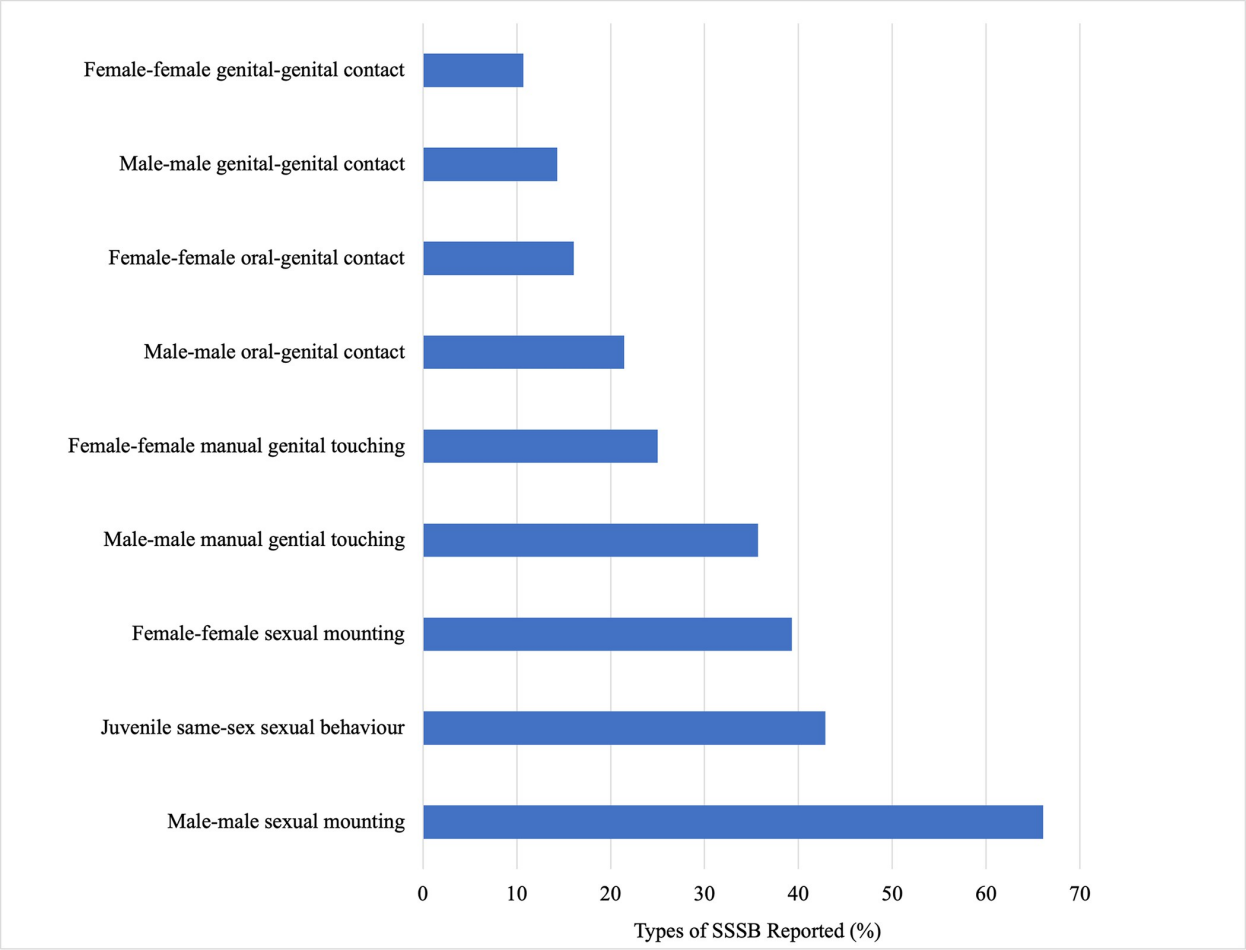
Types of same-sex sexual behaviour observed by respondents in our expert online survey.

Respondents were asked to report on the types of SSSB they had observed in their study species. Male-male sexual mounting was the most frequent behaviour reported (N = 37, 66.1% of reports), along with juvenile same-sex sexual behaviours or play (N = 24, 42.9%), female-female sexual mounting (N = 22, 39.3%), and male-male manual genital touching (N = 20, 35.7%; Fig 2).

Respondents were asked how they recorded observations of SSSB and were able to select multiple answers or use free-form responses in this question. 59.3% (N = 16) of respondents indicated that they recorded SSSB as an affiliative or play behaviour, while 48.2% (N = 13) indicated that they recorded SSSB like other sexual behaviours, 22.2% (N = 6) as a unique behaviour, 11.1% (N = 3) as a dominance or aggressive behaviour, and 7.4% (N = 2) indicated they did not classify SSSB or that they classified it in another way, such as scent marking. Most respondents who collected data on SSSB indicated that they had not published these data (N = 22, 81.5%; Fig 1).

**Fig 3:**
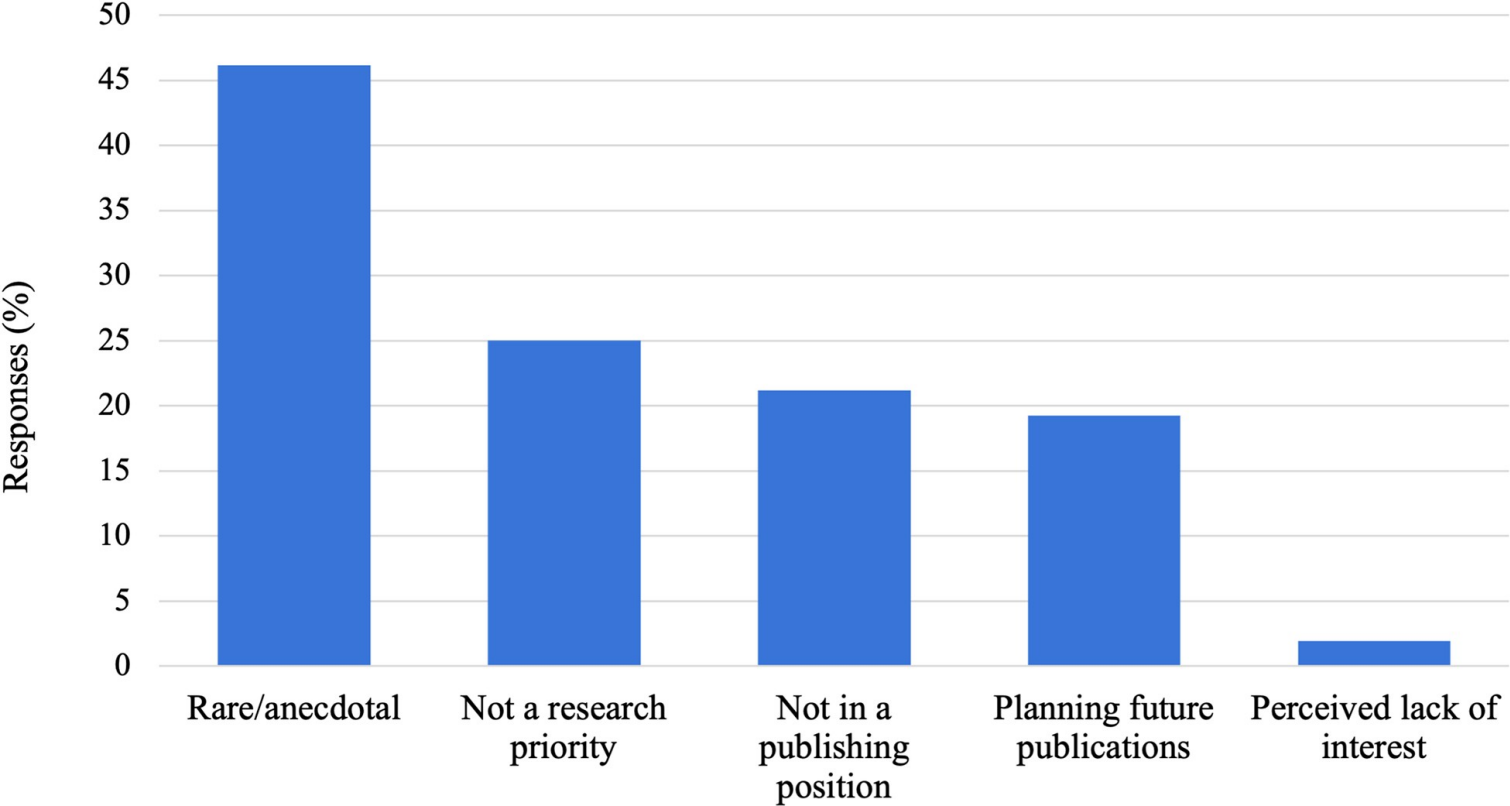
Reasons for not collecting data or publishing on SSSB noted by respondents in our expert online survey.

Respondents were asked to select a reason why they did not collect data on SSSB or why they had not published their data if they did collect it (Fig 3). 46.2% (N = 12) of respondents indicated that SSSB was a rare or anecdotal behaviour in their species, and so there were not enough data to publish. 25.0% (N = 13) of respondents indicated that SSSB did not fit in with, or achieve, the research goals of their lab. 21.2% (N = 11) of respondents indicated that they were not the principal investigator of the lab and therefore did not have control of the research goals or types of publications produced. 19.2% (N = 10) of respondents indicated that although they had not published data on SSSB yet, they planned to do so in the future. 1.9% (N = 1) indicated a perceived lack of interest from editors or journals.

No respondents reported discomfort or sociopolitical concerns at their university or field site as a reason for why they did not collect data or publish on SSSB. When asked in a free form response question to elaborate on their previous response (S1 Text for freeform responses), answers generally fit into one or more of the following six categories: SSSB was not a research interest or priority (N = 16 responses), the behaviour was rare (N = 14), they had plans for, or interest in, a future publication (N = 7), they were unable to collect data on this behaviour (N = 6), they were not in a position to publish or did not have time to publish (N = 5), or the animals they study may exhibit similar behaviours, but these behaviours are not SSSB (e.g., scent gland sniffing) (N = 3).

As a part of our survey, we asked respondents if they self-identified as a member of the LGBTQ+ community. 76.9% (N = 50) of respondents answered No, 20.0% (N = 13) answered Yes, 3.1% (N = 2) selected the “Prefer not to say” option. We considered whether the taxa studied, highest level of education achieved, or self-identification with the LGBTQ+ community predicted whether individuals observed, collected data on, or published on SSSB (Tables 2 and 3).

**Table 2:** Observation, data collection, and publishing on SSSB by those of LGBTQ+ identification in our expert online survey.

Identified as LGBTQ+	Observed SSSB		Collected data on SSSB		Published on SSSB	
	Yes	No	Yes	No	Yes	No
Yes	11	2	6	5	1	5
No	44	13	21	24	4	17
Prefer not to say	1	1	0	1	0	0

**Table 3:** Observation, data collection, and publishing on SSSB by highest education obtained by respondents in our expert online survey.

Education	Observed SSSB		Collected data on SSSB		Published on SSSB	
	Yes	No	Yes	No	Yes	No
Bachelors	5	1	2	3	0	2
Masters	13	4	7	6	1	6
PhD	36	11	18	19	4	14
Did not report	2	1	0	2	0	0

Multiple logistic regressions were performed to assess whether taxa, education level, or identification within the LGBTQ+ community predicted observing, collecting data on, or publishing on SSSB. At an alpha level of 0.05, no models were significant (z = -0.009, p = 0.992, S1 Table; z = -0.337, p = 0.736, S2 Table; z = -0.003, p = 0.998, S3 Table).

## Discussion

Due to our small sample size, the results of this survey should serve as a preliminary analysis of publication trends on SSSB. Most respondents reported on SSSB in Primates, and so the following interpretation of results should be understood as mostly representing the field of primatology, though they may also point to broader trends within all mammalian sciences. Our survey provides preliminary evidence that SSSB occurs more frequently than what is available within the published record. While many of the experts surveyed observed SSSB in their study species, less than half of respondents recorded data on this, and a small percentage of those who recorded data have published on SSSB.

A common theme of the freeform responses was a perceived inability of respondents to do meaningful work on SSSB in their study species. These responses generally fell into three categories, in which respondents indicated that: 1) they were unable to collect data on this behaviour due to the needs of other research priorities, 2) the topics for publication did not ultimately depend on them, or 3) the behaviour was too rare or anecdotal to be considered for publication. One respondent noted when talking about SSSB, “reviewers consider a behaviour with a low expression on the whole and do not consider it to have much relevance” (Table A in S1 Text). From 2010 to 2016, publications of anecdotal reports have decreased in primatology [28]. As the field of animal behaviour advances in the 21^st^ century, methods using quantitative, statistical, and analytical approaches have been favoured over short narrative or anecdotal accounts. This could be due to an assumed lack of scientific vigor in anecdotal reporting or the worry that anecdotal reports leave room for anthropomorphism.

While calls for short reports have increased in some high impact primatological journals recently, and short anecdotal reports continue to be considered important in some schools of thought, for example in Japanese primatology [27], the results of our survey suggest that many mammologists are still wary of publishing anecdotes on SSSB and question the usefulness of such information. Anecdotal reports allow us to compare trends and variability in behaviour across species [105], which improves phylogenetic analyses [106, 107]. Most reports of SSSB occur due to opportunistic observations of SSSB, rather than systematic study of SSSB [1]. Thus, anecdotes are of great importance to the study of SSSB and to understanding its relevance within studies of sexual behaviours more broadly. The availability of anecdotal reports of SSSB, therefore, serves to benefit the scientific community and allows us to better understand the variability and distribution of SSSB across mammals [28].

Our results suggest that expert surveys are a useful tool in wildlife biology, especially in the study of behaviours that are not frequently reported or perceived to be rare. Indeed, we found the use of an expert survey important, and even necessary, given that most respondents have not published their observations of SSSB in their study species. Expert surveys provide us with a tool to access and examine these behaviours on a broader scale [32]. While SSSB was perceived to be rare in frequency by many of our respondents, it was observed by most respondents and appears to be common in many mammals. The results of this study highlight the fundamental role expert surveys play in the analysis of under-published behaviours in many subfields of behavioural ecology.

We hypothesized that social and political factors within universities or field sites, as well as identifying as LGBTQ+, could influence an individual’s reporting of SSSB. However, no respondents reported sociopolitical concerns at their university or field site as a reason for not collecting data on, or publishing on, SSSB in their study species and there was no correlation between identifying as LGBTQ+ and recording data on, or publishing on, SSSB. As we are researchers in WEIRD (Western, Educated, Industrialized, Rich, and Democratic: [108]) countries, it is possible that the reach of our survey was limited to other academics from WEIRD countries who likely face few barriers on the scope of their research topics. A future direction of this work would be to focus surveys on academics from non-WEIRD countries, or the development of a global citizen science initiative, which could include the voices and expertise of scientists across the globe on sexual behaviour in mammals. Mammalian societies in non-WEIRD countries could be contacted, and social networking sites popular in non-English speaking countries can be targeted to further the reach of future surveys.

Additionally, as most (N = 61: 83.6%) respondents studied primates, a disciplinary bias could have resulted in an overreporting of observations of SSSB. Primatologists are often interested in the study of complex social behaviours, and they may be more likely to observe, collect data on, or publish on SSSB than researchers in other fields. In cetaceans, reporting of non-conceptive sexual behaviors was predicted by research effort in each species, but could also be related to the fact that highly social species, who are more likely to engage in non-conceptive sex like SSSB, are also often the research priority [18]. A recent systematic review of SSSB in mammals reported that the majority of SSSB occurred within primates [16]. Thus, while our study sample has a clear bias, SSSB distribution in mammals appears to be biased towards primates regardless. Due to the overrepresentation of primates in our sample, it is possible that our survey findings should only be applicable to the field of primatology, rather than more broadly within mammalian behavioural ecology. When excluding primates from our sample, there still appears to be an underreporting in our small sample size of other mammals. Five of eleven (45.5%) non-primate mammal species reported on in our study did not have existing reports of SSSB in the present literature (Table 1). Future research on this topic should aim to sample mammalian orders evenly to allow for a more comprehensive discussion of reporting trends for SSSB.

As dissemination of the survey involved contacting individuals through social media sites and university listservs, it is likely that our sample population was not random and could have been biased in favour of individuals who have observed SSSB in their study species [109]. Individuals who have not observed SSSB may have chosen to not fill out the survey if they felt they did not have information to contribute to the topic. If individuals did not observe data on SSSB in their study species, they were still asked for taxonomic data on their study species, their education level, and whether they identified as members of the LGBTQ+ community. As such, the results of the multiple logistic regressions could be biased if people who did not observe SSSB chose not to respond to our call for surveys. Only individuals who observed SSSB were asked about data collection and publishing on SSSB, and so the disparity between observations of SSSB and data collection and publication on the topic are still relevant and indicate a potentially significant gap in the publication record on SSSB.

Another possible explanation is that sociosexual behaviours, such as SSSB, could be understudied due to a funding bias. If sociosexual behaviour is not a research priority of funding agencies, researchers who are otherwise interested in studying SSSB may be limited in their ability to do so. Although few survey respondents reported a perceived lack of interest from journals on the topic (N = 1; 1.9%), 25.0% (N = 13) of respondents indicated that SSSB did not fit in with, or achieve, the research goals of their lab. Future work on this topic could explore the proportion of projects on sociosexual behaviour that receive funding by major funding agencies, and how this affects research goals and priorities.

This study demonstrated that same-sex sexual behaviour is observed more frequently than it is published in mammals. We suggest that anecdotal reporting and use of expert surveys can reduce this information gap and allow more research to be conducted on the prevalence and evolutionary significance of SSSB in mammals.

## Supporting information

S1 Appendix Survey questionnaire. (DOCX)

S1 Text Freeform responses. (DOCX)

S1 Table Results of multiple linear regression of the effect of education level, identification within the LGBTQ+ community, and taxa studied on observing SSSB. (DOCX)

S2 Table Results of multiple linear regression of the effect of education level, identification within the LGBTQ+ community, and taxa studied on collecting data on SSSB. (DOCX)

S3 Table Results of multiple linear regression of the effect of education level, identification within the LGBTQ+ community, and taxa studied on publishing on SSSB. (DOCX)
