# Supplementary material for: Same-sex sexual behaviour among mammals is widely observed, yet seldomly reported: Evidence from an online expert survey
Source: PLoS One. 2024 Jun 20;19(6):e0304885. doi: 10.1371/journal.pone.0304885 (PMC11189198; doi:10.1371/journal.pone.0304885)
Supplement: S1 Appendix — (DOCX) [file pone.0304885.s001.docx]

**S1 Appendix.** Survey Questionnaire

This document lists the survey questionnaire used in the study. The survey begins with a consent form:

Consent for Research Participation

Researcher(s): Karyn Anderson, Iulia Badescu, James Gibb, Sergi Lopez-Torres, Malcolm Ramsay, Julie Teichroeb

You are invited to take part in a study to assess the prevalence of same-sex sexual behaviour across mammalian species. Same-sex sexual behaviour includes any sexual interactions between members of the same-sex. This could include mounting, genital touching, oral-genital contact, and juvenile sexual behaviour or play. We are interested in recording both the presence of same-sex sexual behaviour in your species, or the absence. The box below highlights key information about this research for you to consider when making a decision whether or not to participate. Carefully consider this information and the more detailed information provided below the box. Please ask questions about any of the information you do not understand before you decide whether to participate.

Key Information for You to Consider

Voluntary Consent: You are being asked to volunteer for a research study. It is up to you whether you choose to participate or not. There will be no penalty or loss of benefits to which you are otherwise entitled if you choose not to participate or discontinue participation.

Purpose: The aim of this survey is to determine which mammal species exhibit same-sex sexual behaviour but have not been published on, and to determine why individuals may not be reporting this information in their study species.

Duration: It is expected that your participation will last 10-15 minutes during the survey.

Procedures and Activities: You will be asked to participate in an online survey on same-sex sexual behaviour in your study species. This survey will ask you to identify your species, whether or not they engage in same-sex sexual behaviour, if you have published on this data, and possible reasons why if you have not published on this data.

Potential Risks: This study includes an optional question asking participants if they identify broadly as LGBTQ+. We recognize that this question may cause discomfort for some participants. If you do not want to include this information, you can opt out of this question.

Confidentiality: All information collected as part of this research project will remain confidential within the limits provided by the law. You are participating in this study confidentially. We will not use your name or any information that would allow you to be identified in any publications or presentations. All participants will be assigned an ID number, rather than your real name. Study investigators will be the only persons with access to the raw data for this project. Survey results will be stored on a secure, password-protected server (Sharepoint and OneDrive) and will be available to the research team for analysis. Electronic files (notes) will be password-protected and encrypted on Study investigators computers. Study ID numbers and descriptions will be kept separate from data.

Participation and Withdrawal: You may withdraw your consent to participate in any portion of the study at any time by contacting the PI until data analysis begins in August 2021. At that time, results will be distributed, and consent can no longer be withdrawn.

Costs: There are no financial costs to you participating in the study.

Information about the Study: We expect to have preliminary results by August of 2021. If you are interested in receiving a summary of results or any material produced from this research please contact the study investigators.

Questions: For questions regarding this study, please contact Karyn Anderson (karyn.anderson@mail.utoronto.ca).

For questions about your rights as a research participant, you may contact The University of Toronto’s Office of Research Oversight and Compliance at 416-946-3273, or at [ethics.review@utoronto.ca](mailto:ethics.review@utoronto.ca)

Do you consent to these terms? Clicking on the “Yes” button below will start the survey and confirm your consent to participate in this study.

- 1. Yes  *Skip to question 1*
  2. No *End survey*

Study Species Information

1. What is your study species? (scientific name). If you research multiple species and would like to include information on same-sex sexual behaviour in more than one species, please fill out a new survey for each species.
   1. (Blank field)
2. Where is your field site? (Town/City, Country)
   1. (Blank field)
3. Have you observed your study species engaging in same-sex sexual behaviour? Same-sex sexual behaviour is defined as any sexual interactions between members of the same-sex. This could include mounting, genital touching, oral-genital contact, juvenile sex play behaviours, or others.
   1. Yes
   2. No *Skip to question 12*

Same-sex sexual behaviours

1. Please select any of the same-sex sexual behaviours which apply to your study species:
   1. Male-male sexual mounting (including thrusting)
   2. Female-female sexual mounting (including thrusting)
   3. Male-male genital touching (outside of a grooming context)
   4. Female-female genital touching (outside of a grooming context)
   5. Male-male oral-genital contact
   6. Female-female oral-genital contact
   7. Juvenile same-sex sexual behaviour/play
   8. Other (specify): (blank field)
2. Do you collect data on this behaviour?
   1. Yes *Skip to question 6*
   2. No *Skip to question 10*
3. What methods do you use?
   1. (Blank field)
4. How do you classify this behaviour?
   1. Same as other sexual behaviour (i.e. copulation/mounting/etc.)
   2. As a dominance or aggressive behaviour
   3. As an affiliative behaviour
   4. Other: (blank field)
5. Have you published any data on same-sex sexual behaviour in your species?
   1. Yes *Skip to question 9*
   2. No *Skip to question 10*
6. Can you recommend the source?
   1. (Blank field) *Skip to question 13*

SSSB Reporting

1. Please select a reason as to why you have not collected data on and/or published on this information:
   1. Not enough data because behavior too rare/anecdotal
   2. Not enough data yet, but planning on publishing in the future
   3. I am not the PI at this site/not responsible for publishing results
   4. Does not fit with or achieve the research goals of our lab
   5. Perceived lack of interest from editors/journals
   6. Socio-political concerns (i.e. homophobic norms at fieldsite or home institution)
   7. Discomfort at reporting/publishing this type of behaviour
   8. Other (specify below)
2. Please elaborate on the reason listed above for not publishing
   1. (Blank field)
3. Can you recommend other source(s) reporting SSSB in your study species?
   1. (Blank field)

Additional Information

1. Is there additional information you would like to include in your response?
   1. (Blank field)
2. Education (please provide information on terminal degree, and field(s))
   1. (Blank field)
3. Current Employment/Occupation (for student respondents, please note whether you are currently an undergraduate or graduate student)
   1. (Blank field)
4. In this study, we are interested in whether LGBTQ+ individuals are more likely to report on same-sex sexual behaviour in their study species. You can opt out of this question by selecting "Prefer not to say." Do you self-identify broadly as a member of the LGBTQ+ community?
   1. Yes
   2. No
   3. Prefer not to say
5. Would you be interested in being contacted for future phases of this project (e.g. interviews?)
   1. Yes *Skip to question 18*
   2. No *End survey*
6. Here is my name and contact information
   1. (Blank field)
