# Supplementary material for: Same-sex sexual behaviour among mammals is widely observed, yet seldomly reported: Evidence from an online expert survey
Source: PLoS One. 2024 Jun 20;19(6):e0304885. doi: 10.1371/journal.pone.0304885 (PMC11189198; doi:10.1371/journal.pone.0304885)
Supplement: S1 Text — (DOCX) [file pone.0304885.s002.docx]

**S2 Text.** Freeform Responses

The following freeform responses are in response to questions about why the survey participants did not collect data on, or publish collected data, on SSSB in their study species. The freeform responses have been classified (Table A). Some responses fit multiple categories and were included as such. Identifying information of freeform responses has been censored to avoid jeopardizing the anonymity of survey participants.

**Table A. Classification of Freeform Responses**

| Not a research interest | 16 |
| --- | --- |
| Was not/can not collect data on this, may be missing this behaviour if it occurs OOS | 6 |
| Too rare of a behaviour/reviewers consider it too rare | 14 |
| Planning a future publication or data collection, or possibly interested in the future | 7 |
| Not in a publishing position (ex. Undergrad), haven’t had time or don’t have time to publish | 5 |
| May exhibit similar behaviours but it’s a part of another behaviour, ex. Scent gland sniffing, affiliative or dominance behaviours | 3 |

**Freeform responses:**

- I don't study behaviour.
- Not enough data and collected opportunistically. I collected ad lib data since what I was seeing seemed worth recording, but this was not part of the data collection used for my ongoing project at that site, so I haven't had opportunity to publish it or report on it.
- Behaviour observed during ongoing data collection for a long-term project, and our data collection and ethogram is not set up to record nuances of social behaviour such as the details of sexual behaviour
- SSSBs have never been a priority for our research group and so we haven't emphasized that our field assistants (who are responsible for most of the collected data) should record these behaviours. In my opinion, it is not that our group is not interested in these behaviours, but rather that we don't want to assign too much work to our field assistants who are already collecting a lot of behavioural data. They have recently started to use iPads to collect data and I think that with this new tool, it will be easier to record and manage anecdotal data like this. Definitely interested in the topic with orangutans and other primates, but the occurrences are infrequent. No hesitation to discuss or engage in research on this topic. Happy to assist in the future.
- This behaviour is not common enough nor the focus of my current research program
- Elaborating a manuscript
- Not the topic of my studies, so unfortunately there is no time to study this in more detail.
- Witnessed behaviour as undergraduate student and as care staff rather than in a publishing position
- Still compiling similar data for future publication.
- We've seen female-female mounting 3 times in 40 yrs; and male-male mounting only twice. None of this involved genital stimulation - they just went through the motions.
- Behavior occurs rarely and does not seem particularly interesting.
- Mounting and presenting is part of baboons' affiliative/dominance behavioural repetoire along with other behaviours such as lip-smacking and 'come-here' faces.
- Mongooses have scent glands around their anus so genital contact is part of scent-marking behaviour (they scent mark each other)
- I will have to read more about this behavior. This behavior might not appear all the time, or we might miss recording it if the individuals are playing out of sight or in dense bushes. I am also not convinced on publishing about it until I am sure about where the literature guides me and the conclusions I can draw on the basis of existing knowledge.
- I think most of the behaviours are well described in chimpanzees, but it may be interesting to look at frequencies in different contexts in the future.
- I am not looking at sexual behaviors in my research
- I just defended my master's degree and I still haven't been able to publish the data, this happens especially because the reviewers consider a behavior with a low expression on the whole and do not consider it to have much relevance
- Infrequent anecdotal behaviour is hard to use to test hypotheses and hard to publish
- No time yet
- This behaviour is not seen very often. We have a few records. We hope to explore this in the future.
- Same-sex sexual behaviour was not relevant to any of my previous reseach questions
- Not part of the study- but noted as ad lib for focal subjects (females only). Observed in male-male contexts most often.
- Incidental observation while in the field following siamang
- It was too infrequent and I need a proper social context paper to publish these data.
- So so so rarely observed that it did not register in analyses
- In rhesus groups we observe that Juveniles often show same sex sexual mounting during social interaction. Grooming of the genital parts is sometimes observed in my group mainly among adult females.
- i have not collected information systematically, the data are rare
- I do collect some data regarding same sex behavior of the Long-tailed macaque, but very small portion and not enough to publish. At the time it was out of my interest. Lately i realized that this behavior is interesting, and factor motivated individuals behave like that still not well known. I need to menioned the LTM of my research is highly provisioning group.
- Have only seen once or twice
- This is not my PIs field of study
- Only observed once
- I collect data on mammal community composition using camera-traps, and have occasional photographic records of same-sex incidents between male pig-tailed macaques (Macaca nemestrina). Personally I find this interesting, but behavioural questions are not currently the focus of my research. It may be of interest that of 57 mammal species recorded, M. nemestrina are the only species so far with confirmed photographic records of same-sex interactions.
- I only observed this behavior twice. I have mentioned it in published material, but do not have enough data for a free-standing publication.
- I don't have the time to collect the data on this behavior. It's interesting but a low priority.
- I do annotate any event that I see, but it only occurs occasionally.
- Neurobiological research took priority for research program survival. However, the monkeys (N=30 adult females and N=14 adult males) are now retired and live at a sanctuary (***) and we can now conduct behavior studies.
- "it wasn't the focus of my research at that time and the responsible of the site was not interesting in publish this kind of observation at time. But in the past, 2002, it was already publish in a congress abstract:
  - ***
- Was not investigated systematically during time period in which observed 1991-2, then grant expired, I no longer work at Yerkes, and have been at at teaching school that allows little time for research ever since.
