# Supplementary material for: Same-sex sexual behaviour among mammals is widely observed, yet seldomly reported: Evidence from an online expert survey
Source: PLoS One. 2024 Jun 20;19(6):e0304885. doi: 10.1371/journal.pone.0304885 (PMC11189198; doi:10.1371/journal.pone.0304885)
Supplement: S1 Table — (DOCX) [file pone.0304885.s003.docx]

| **S3 Table. Results of multiple linear regression of the effect of education level, identification within the LGBTQ+ community, and taxa studied on observing SSSB** | | | | |
| --- | --- | --- | --- | --- |
|  | Estimate | Std. Error | z value | Pr(>\|z\|) |
| (Intercept) | -13.761621 | 1486.02503 | -0.0092607 | 0.9926111 |
| Masters | -0.1811566 | 1.3563135 | -0.1335654 | 0.8937462 |
| PhD | -0.1762733 | 1.2327832 | -0.1429881 | 0.8862996 |
| LGBTQ+ Yes | 0.564651 | 0.8864658 | 0.6369687 | 0.5241452 |
| Artiodactyla | 17.1096403 | 4612.19537 | 0.0037097 | 0.9970401 |
| Carnivora | 17.6718496 | 2917.01268 | 0.0060582 | 0.9951663 |
| Proboscidea | 17.5885362 | 4608.24892 | 0.0038168 | 0.9969547 |
| Rodentia | 17.6751051 | 3765.84232 | 0.0046935 | 0.9962551 |
