# Supplementary material for: Same-sex sexual behaviour among mammals is widely observed, yet seldomly reported: Evidence from an online expert survey
Source: PLoS One. 2024 Jun 20;19(6):e0304885. doi: 10.1371/journal.pone.0304885 (PMC11189198; doi:10.1371/journal.pone.0304885)
Supplement: S2 Table — (DOCX) [file pone.0304885.s004.docx]

| **S4 Table. Results of multiple linear regression of the effect of education level, identification within the LGBTQ+ community, and taxa studied on collecting data on SSSB** | | | | |
| --- | --- | --- | --- | --- |
|  | Estimate | Std. Error | z value | Pr(>\|z\|) |
| (Intercept) | -0.2208748 | 0.6403267 | -0.3449407 | 0.7301389 |
| Masters | 0.2775535 | 0.7196756 | 0.3856647 | 0.699745 |
| PhD | 0.2749735 | 0.6539055 | 0.4205096 | 0.6741132 |
| LGBTQ+ Yes | -0.1376034 | 0.4846088 | -0.2839473 | 0.7764507 |
| Artiodactyla | 5.2930761 | 266.40359 | 0.0198686 | 0.9841482 |
| Carnivora | -0.89572 | 0.6848225 | -1.3079593 | 0.1908871 |
| Proboscidea | 0.082098 | 0.9776959 | 0.0839709 | 0.9330795 |
| Rodentia | 0.3750895 | 0.8069626 | 0.4648164 | 0.642063 |
