# Supplementary material for: Same-sex sexual behaviour among mammals is widely observed, yet seldomly reported: Evidence from an online expert survey
Source: PLoS One. 2024 Jun 20;19(6):e0304885. doi: 10.1371/journal.pone.0304885 (PMC11189198; doi:10.1371/journal.pone.0304885)
Supplement: S3 Table — (DOCX) [file pone.0304885.s005.docx]

| **S5 Table. Results of multiple linear regression of the effect of education level, identification within the LGBTQ+ community, and taxa studied on publishing on SSSB** | | | | |
| --- | --- | --- | --- | --- |
|  | Estimate | Std. Error | z value | Pr(>\|z\|) |
| (Intercept) | -18.56607 | 6522.6386 | -0.0028464 | 0.9977289 |
| Masters | 17.46746 | 6522.63871 | 0.002678 | 0.9978633 |
| PhD | 17.46746 | 6522.63864 | 0.002678 | 0.9978633 |
| LGBTQ+ Yes | 0 | 1.333333 | 0 | 1 |
| Artiodactyla | -17.46746 | 4612.20218 | -0.0037872 | 0.9969782 |
| Carnivora | -17.46746 | 6522.63864 | -0.002678 | 0.9978633 |
| Proboscidea | 0 | 9224.40397 | 0 | 1 |
| Rodentia | -17.46746 | 4612.20213 | -0.0037872 | 0.9969782 |
